# Falls in hospitalized older adults and the use of fall risk-increasing drugs and anticholinergic medications in Colombia: a case‒control study

**DOI:** 10.3389/fphar.2024.1369200

**Published:** 2024-07-03

**Authors:** Manuel E. Machado-Duque, Lina Camacho-Arteaga, Mónica Sabaté, Xavier Vidal-Guitart, Jorge E. Machado-Alba

**Affiliations:** ^1^ Departmento de Farmacología, Terapeutica y Toxicología, Universitat Autònoma de Barcelona, Bellaterra, Spain; ^2^ Grupo de Investigación en Farmacoepidemiologia y Farmacovigilancia, Universidad Tecnológica de Pereira-Audifarma SA, Risaralda, Colombia; ^3^ Grupo de Investigación Biomedicina, Facultad de Medicina, Fundación Universitaria Autonoma de las Americas, Pereira, Colombia; ^4^ Departmento de Farmacología Clinica, Hospital Universitari Vall d'Hebron, Hospital Campus Vall d'Hebron Barcelona, Barcelona, Spain; ^5^ Grupo de Investigación en Farmacología Clínica, Vall d'Hebron Institut de Recerca (VHIR), Vall d'Hebron Hospital Universitari, Barcelona, Spain

**Keywords:** elderly aged, falls, fall risk-increasing drugs, hospital inpatients, cholinergic antagonists

## Abstract

**Introduction:**

In-hospital falls are multicausal in older hospitalized patients. Drugs with anticholinergic load and psychotropic effects can increase the risk of falling.

**Objective:**

This study aimed to determine the associations between fall risk-increasing drugs (FRIDs) and the anticholinergic risk score (ARS) with falls in hospitalized older hospitalized patients.

**Methods:**

This was a case‒control study of patients ≥65 years of age of either sex treated in four clinics in Colombia between 2018 and 2020. Each patient who suffered a fall during hospitalization was matched with four hospitalized patients who did not. Sociodemographic, clinical, and pharmacologic variables and the use of the ARS and FRIDs were evaluated. The risk associated with FRIDs was estimated using conditional logistic regression.

**Results:**

There were 250 patients and 1,000 controls (ratio of 1:4), with a mean age of 77.4 ± 7.4 years and a predominance of men (*n* = 800, 64.0%). The majority of falls occurred during hospitalization (*n* = 192 patients, 76.8%). Polypharmacy, calcium channel blockers, antiepileptics, antipsychotics, sodium–glucose cotransporter type 2 inhibitors, and nonsteroidal anti-inflammatory drugs were associated with falls during hospitalization. With an ARS score of 3, the probability of falling during the hospital stay increased (aOR: 2.34; 95% CI: 1.64–3.32).

**Conclusion:**

There is an association between suffering a fall and the use of drugs with anticholinergic load or FRIDs in hospitalized adults more than 65 years of age in Colombia.

## Introduction

A fall is a definite event in which a person loses balance and ends up on the floor or some firm ground. It is more prevalent in the population aged ≥65 years, in which the risk of falls has been estimated at 27.1%–34.8% of the total reported security incidents, making it a public health problem with a significant impact on hospitalized patients. This common adverse event reported in hospitals can result in multiple complications, ranging from minor blows or trauma to severe injuries and death (Healey et al., 2008; Anderson et al., 2016).

Different factors in older adults in the outpatient setting can increase the risk of falls, such as living alone, a decrease in instrumental capacity, previous falls, low blood pressure, weight deficit, cognitive impairment, and the use of drugs with anticholinergic load or others that increase the risk of falls [fall risk-increasing drugs (FRIDs)] (Machado-Alba et al., 2016; Machado-Duque et al., 2017; Zia et al., 2017; Andersen et al., 2020; Seppala et al., 2021). Male sex, history of hip replacement, previous falls, frailty, confusion, alterations in balance, and use of psychotropic medications or FRIDs are risk factors for falls in hospitalized patients (Krauss et al., 2005; Aryee et al., 2017; Castaldi et al., 2022; Ramos et al., 2023). There are few studies on medication use and falls in older adults in the hospital context or on the differences in the estimated risk of falls caused by different medications with psychotropic and sedative capacities nor are there many assessments of the association between falls and the anticholinergic risk scale (ARS) and FRIDs. Studies of hospital falls have focused mainly on the risks associated with the caregiver and nursing staff, as well as the comorbidities themselves or external factors, without clearly identifying the risk associated with medications and how these can be predictors of worse outcomes (Anderson et al., 2016).

The ARS was devised in 2008 by Rudolph et al. (2008). They hypothesized that drugs with anticholinergic potential are associated with falls or delirium, and they constructed a categorical scale in which the highest scores are associated with a greater probability of adverse events. The FRID scale proposes that the use of some psychotropic medications, such as opioids, antipsychotics, anxiolytics (benzodiazepines), hypnotics/sedatives, and antidepressants, increases the risk of falls, with greatest risk arising when a patient takes two or more drugs from this group (HR: 1.67; 95% CI: 1.58–1.77; Laflamme et al., 2015).

This association between the use of potentially inappropriate medications and falls in older adults is stronger in the presence of more comorbidities, which results in the prescription of more medications (Johnell and Fastbom, 2008) and increases the probability of pharmacologic interactions and other problems related to medications. A correct pharmacotherapeutic evaluation of a hospitalized patient, especially a patient aged ≥65 years and with polypharmacy, can identify inappropriate or risky prescriptions and can reveal the probability of suffering a fall and the potential need to avoid particular higher-risk medications (Maly et al., 2019).

Falls in hospital patients have multiple causes, potentially including risky medications. Additionally, available literature on risk factors is sparse, and no study on this topic has been carried out in Colombia. We aimed to establish the associations between the use of drugs according to the FRID classification and their anticholinergic load and the occurrence of falls in adults aged ≥65 years during their hospitalization at four hospital centers in Colombia from 2017 to 2020.

## Materials and methods

### Participants

A case‒control study was carried out in patients aged ≥65 years of either sex, who were treated between 1 January 2018 and 31 December 2020 in emergency services and were hospitalized in four Colombian clinics in the cities of Armenia, Cali, Pereira, and Popayán (central-western Colombia, with a mainly urban area of influence with greater average development) between 1 January 2018 and 31 December 2020. As of December 2020, the four centers had 855 beds available and treated a total of 530,097 patients over 4 years.

Those who had a fall during the hospital stay in the study period were selected for the case group. Four hospitalized patients were matched to each patient (ratio 1:4). The matching criteria were sex, age within 2 years, hospitalization within a month of the patient’s fall, the same main pooled diagnosis at admission (both the case and his four controls were admitted for the same diagnosis: for example, community-acquired pneumonia, acute coronary event, and exacerbation of heart failure), and not falling during their hospital stay.

### Sample size

It was expected to have at least 1,200 participants due to an expected prevalence of psychotropic use with a risk of falls of 67.7% and 58.3% in the controls and an expected OR of higher risk of 1.5 (Machado-Duque et al., 2017). We required a power greater than 80% and an alpha error of 0.05. Therefore, a sample of 250 cases and 1,000 controls (1:4 ratio) met these requirements.

### Data source

The safety records of adverse events of each clinic were taken as a source of information for the identification of cases in which those who suffered falls and the reasons for this were regularly monitored (mandatory notification). Patients with incomplete information in the registry were excluded. For the other variables, the medical records of each patient and each control were evaluated, and for the evaluation of the drugs used in the research participants, the dispensing records were obtained during hospitalization from the electronic database of the logistics operator Audifarma SA (Franco and Vizcaya, 2020), which supplied them. This drug dispensing manager has a database of claims for drugs and other supplies of almost 9.5 million Colombians, both ambulatory and hospital deliveries, which has been used in multiple pharmacoepidemiological studies.

The following groups of variables were gathered:

1. Sociodemographic data included age, sex, city of residence, and health insurer status.
2. Clinics: main clinical diagnosis at admission, date of emergency care, or admission to hospitalization.
3. Comorbidities: pathologies reported in medical records, with special emphasis on alterations in the state of consciousness, vertigo, arterial hypertension, diabetes mellitus, and others reported.
4. The pharmacologic group included drugs used during hospitalization, with a special emphasis on those with anticholinergic load, psychoactive drugs such as anticonvulsants, antipsychotics, benzodiazepines, and antidepressants. Each drug was identified by a unique code related to the logistic operator’s database by molecule and presentation.

The drugs used were classified for analysis following the FRID classification and ARS classification.

5. FRIDs taken: antihypertensives included in the FRID classification: loop diuretics and α1 antagonists. Psychotropics included in the FRID classification include antipsychotics, antidepressants, and benzodiazepines. Other FRIDs include anticonvulsants, opioids, NSAIDs, and antihistamines (Seppala et al., 2021).
6. ARS: The anticholinergic load was calculated with the ARS (Rudolph et al., 2008) (Supplementary Table S1) for each drug prescribed to patients or controls. Each patient’s individual drug ARS scores were summed to yield the following classifications: 0 points (no risk), 1 point (low risk), 2 points (high risk), and ≥3 points (very high risk) (Rudolph et al., 2008).

### Statistical analysis

The collected variables were recorded in a Microsoft Excel spreadsheet with closed fields. Univariate analyses are presented as comparisons of frequencies and proportions for categorical variables and of measures of central tendency, position, and dispersion for quantitative variables, according to their normality (Kolmogorov‒Smirnov test). For bivariate analyses, Student’s t-test was used to compare quantitative variables, and the *χ* ^2^ test was used for categorical variables. Crude OR and adjusted OR point estimates were calculated, along with their 95% CI; *p* < 0.05 was the threshold for statistical significance.

Conditional logistic regression multivariate analysis models were constructed in which ORs were adjusted for the confounders identified in the previous bivariate analysis (*χ* ^2^) that showed statistically significant differences, along with other variables that could feasibly explain the outcome. Three different multivariate logistic regression models adjusted for comorbidities were used. In the first model, the exposure variable was all the drugs previously associated with the covariates and comorbidities. In the second model, the exposure was classified by the ARS score (reference category 0). A third model compares the classification of FRID drug groups (mutually exclusive) for each FRID medication grouping variable, with the reference variable not having medications from this group for each analysis. The ORs adjusted for the associated comorbidities in the bivariate analysis, *p* values, and 95% confidence intervals are presented. For the statistical computations, the statistical package SPSS for Windows version 28.0 was used (IBM, USA).

## Results

A total of 250 older hospitalized patients with one or more falls were analyzed from the included hospitals in Colombia during the study period. Four controls for each patient were included, for a total of 1,000 controls. The mean age of all patients (cases and controls) was 77.4 ± 7.4 years. Men predominated overall (*n* = 800, 64.0%) and in both groups.

The main reason for admission of patients and controls was community-acquired pneumonia (12.4% and 12.5%, respectively), followed by decompensated heart failure (10.8% and 10.5%), with no significant differences. No differences were found in the frequencies by sex or admission diagnoses between cases and controls, nor in the mean age. The most frequent comorbidities found were arterial hypertension (64.8% of patients and 64.3% of controls, *p* = 0.134) and a history of coronary disease (16.4% of patients and 30.2% of controls, *p* < 0.001). For most of the concomitant diseases, there were no significant differences between the groups, except for delirium, vertigo, coronary disease, and cerebrovascular events. Table 1 shows the sociodemographic variables, admission diagnoses, and comorbidities of the patients and controls, as well as the bivariate comparisons for each category.

**TABLE 1:** Sociodemographic variables, admission diagnoses and comorbidities of cases and controls, and frequency and bivariate comparison in older hospitalized patients in four hospitals in Colombia.

Variable	Cases with fall (*n* = 250)	Control (*n* = 1000)	*p*-value
Sociodemographic			
Age in years (mean, *SD*)	77.4 (7.4)	77.4 (7.6)	0.93
Male sex—No (%)	160 (64.0)	640 (64.0)	1.000
Age group—No (%)			
65–74 years	102 (40.8)	393 (39.3)	0.912
75–84 years	108 (43.2)	411 (41.1)	0.922
+85 years	40 (16.0)	240 (19.6)	0.923
Admission diagnoses			
Community-acquired pneumonia	31 (12.4)	125 (12.5)	0.966
Heart failure	27 (10.8)	105 (10.5)	0.890
Cancer	25 (10.0)	101 (10.1)	0.963
Infection cause^a^	26 (10.4)	97 (9.7)	0.740
Coronary heart disease	20 (8.0)	84 (8.4)	0.838
COVID-19	21 (8.4)	84 (8.4)	1.000
Cerebrovascular event	15 (6.0)	61 (6.1)	0.953
Major surgery	14 (5.6)	58 (5.8)	0.903
Urinary tract infection	14 (5.6)	55 (5.5)	0.951
Fracture	14 (5.6)	54 (5.4)	0.901
Cognitive impairment	6 (2.4)	26 (2.6)	0.897
Bleeding	6 (2.4)	23 (2.3)	0.925
Renal disease	6 (2.4)	22 (2.2)	0.848
Diabetic foot	4 (1.6)	16 (1.6)	1.000
Cirrhosis	3 (1.2)	10 (1.0)	0.780
Other diagnoses	18 (7.2)	79 (7.9%)	0.711
Region			
Pereira, Risaralda	103 (41.2)	412 (41.2)	1.000
Cali, Valle del Cauca	77 (30.8)	308 (30.8)	
Popayán, Cauca	45 (18.0)	180 (18.0)	
Armenia, Quindío	25 (10.0)	100 (10.0)	
Comorbidities			
Hypertension	162 (64.8)	643 (64.3)	0.134
Coronary disease	41 (16.4)	302 (30.2)	<0.001
Chronic obstructive pulmonary disease	51 (20.4)	232 (23.2)	0.344
Type 2 diabetes mellitus	64 (25.6)	224 (22.4)	0.283
Heart failure	45 (18.0)	212 (21.2)	0.263
Chronic kidney disease (GFR < 60 mL/min)	54 (21.8)	167 (16.7)	0.061
Active cancer	26 (10.4)	137 (13.7)	0.166
Hypothyroidism	35 (14.0)	125 (12.5)	0.525
Dyslipidemia	21 (8.4)	114 (11.4)	0.172
Delirium	49 (19.6)	99 (9.9)	<0.001
Stroke or transient cerebral ischemia	11 (4.4)	87 (8.7)	0.024
Dementia	16 (6.4)	61 (6.1)	0.860
Rheumatologic disease	10 (4.0)	37 (3.7)	0.824
Dizziness	10 (4.0)	14 (1.4)	0.007
Epilepsy or seizure episodes	3 (1.2)	14 (1.4)	0.807
Parkinson’s disease	3 (1.2)	13 (1.3)	0.900
Urinary incontinence	6 (2.4)	12 (1.2)	0.154
Depression/anxiety	6 (2.4)	11 (1.1)	0.112

^a^ Infection cause: Infectious diseases other than urinary tract infection, community-acquired pneumonia, and diabetic foot.

Most of the falls occurred during hospitalization (*n* = 192 patients, 76.8%). Most often, the falls were related to the process of standing alone on the way to the bathroom without the help of a family member or nursing staff (*n* = 113, 45.2%).

The most prescribed drug groups during hospitalization were antihypertensives (88.0% in cases and 75.1% in controls), statins (62.0% in cases and 51.1% in controls), acetylsalicylic acid and other antiplatelet agents, proton pump inhibitors, antipyretic analgesics, NSAIDs, opioids, and psychotropics. For almost half of the groups of drugs identified, statistically significant differences were found in the frequency between cases and controls, including some antihypertensive, lipid-lowering, first-generation antihistamines, antidiabetics, antiparkinsonians, anticonvulsants, typical and atypical antipsychotics, benzodiazepines, antidepressants, opioid and nonopioid analgesics, and even disease-modifying antirheumatic drugs. Table 2 shows these groups and their differences in bivariate comparisons.

**TABLE 2:** Medications prescribed in cases of falls and controls, and frequency and bivariate comparison in older hospitalized patients in four hospitals in Colombia.

Cardiovascular use	Cases with fall (*n* = 250)	Control (*n* = 1000)	*p*-value
Antihypertensives			
* Angiotensin receptor blockers*	148 (59.2)	478 (47.8)	0.001
* Loop diuretics*	142 (56.8)	418 (41.8)	<0.001
* Calcium antagonists*	123 (49.2)	352 (35.2)	<0.001
*Beta blockers*	119 (47.6)	439 (43.9)	0.293
* Angiotensin-converting enzyme inhibitors*	62 (24.8)	277 (27.7)	0.356
* Mineralocorticoid receptor antagonists*	52 (20.8)	169 (16.9)	0.148
* Thiazides*	37 (14.8)	141 (14.1)	0.777
* Alpha 1 and central blockers*	35 (14.0)	117 (11.7)	0.320
* Central origin antihypertensives*	35 (14.0)	76 (7.6)	0.001
* Angiotensin receptor–neprilysin inhibitor*	5 (2.0)	8 (0.8)	0.940
Others for cardiovascular use			
* Atropine*	41 (16.4)	131 (13.1)	0.175
* Other antiarrhythmics*	27 (10.8)	109 (10.9)	0.964
* Methyldigoxin*	8 (3.2)	25 (2.5)	0.537
* Selective alpha 1 blockers*	0	0	
Lipid-lowering drugs			
* Statins*	155 (62.0)	511 (51.1)	0.002
* Fibrates*	2 (0.8)	4 (0.4)	0.345
* Ezetimibe*	0	0	NA
*Hematic system and others*			
* Acetylsalicylic acid*	114 (45.6)	419 (41.9)	0.290
* Clopidogrel and other antiplatelet agents P2Y12i*	60 (24.0)	240 (24.0)	1.000
* Direct oral anticoagulants*	14 (5.6)	45 (4.5)	0.463
* Warfarin*	1 (0.4)	15 (1.5)	0.166
Digestive, respiratory, and endocrine use			
Digestive			
* Proton pump inhibitors*	182 (72.8)	677 (67.7)	0.120
* Hyoscine butylbromide*	87 (34.8)	287 (28.7)	0.060
* Anti-H2 (ranitidine)*	4 (1.6)	27 (2.7)	0.317
Respiratory (at least one)			
* Inhaled anticholinergics*	100 (40.0)	344 (34.4)	0.098
* B2 agonists*	78 (31.2)	306 (30.6)	0.854
* Theophylline*	3 (1.2)	7 (0.7)	0.427
Antihistamines			
* First-generation antihistamines*	29 (11.6)	65 (6.5)	0.006
* Second-generation antihistamines*	10 (4.0)	41 (4.1)	0.943
Endocrinological			
* Levothyroxine*	34	13.7	
* *Any antidiabetic	57	22.9	
* Fast-acting insulin*	46 (18.4)	11 (11.0)	0.002
* Long-acting insulin*	38 (15.2)	87 (8.7)	0.002
* Metformin*	20 (8.0)	78 (7.8)	0.916
* DPP-4 inhibitors*	10 (4.0)	16 (1.6)	0.025
* SGLT-2 inhibitors*	5 (2.0)	5 (0.5)	0.032
* Sulfonylurea*	0 (0.0)	3 (0.3)	0.386
* GLP-1 receptor agonists*	0	0	NA
Antiparkinsonians			
* Pyridostigmine/neostigmine*	13 (5.2)	48 (4.8)	0.793
* Biperiden*	3 (1.2)	0 (0.0)	0.001
* Levodopa*	3 (1.2)	13 (1.3)	0.900
Use in the central nervous system, inflammation, and pain			
Any psychotropic drug			
* Antiepileptics*	63 (25.2)	118 (11.8)	<0.001
* Conventional antipsychotics*	63 (25.2)	108 (10.8)	<0.001
* Memantine*	5 (2.0)	5 (0.5)	0.017
* Atypical antipsychotics*	36 (14.4)	43 (4.3)	<0.001
* Amantadine*	2 (0.8)	3 (0.3)	0.263
* Benzodiazepines*	99 (39.6)	288 (28.8)	0.001
* Any antidepressant*			
*Trazodone*	42 (16.8)	108 (10.8)	0.009
* Selective serotonin reuptake inhibitors*	34 (13.6)	64 (6.4)	<0.001
* Atypical*	4 (1.6)	8 (0.8)	0.246
* Tricyclics*	4 (1.6)	17 (1.7)	0.912
* Selective serotonin + norepinephrine reuptake inhibitors*	2 (0.8)	4 (0.4)	0.345
* Any analgesic*			
* Nonsteroidal anti-inflammatory drugs*	111 (44.4)	302 (30.2)	<0.001
* Acetaminophen*	175 (70.0)	558 (55.8)	<0.001
* Dipyrone*	84 (33.6)	328 (32.8)	0.810
*Any opioid*			
* Partial agonist opioids*	149 (59.6)	441 (44.1)	<0.001
* Full agonist opioids*	132 (52.8)	444 (44.4)	0.017
* Z drugs*	1 (0.4)	0 (0.0)	0.450
Inflammation and others			
* Systemic corticosteroids*	99 (39.6)	334 (33.4)	0.650
* Conventional DMARDs*	7 (2.8)	8 (0.8)	0.009
* Biological DMARDs*	1 (0.4)	1 (0.1)	0.288
* Antimigraine drugs*	0	0	

DPP-4, dipeptidyl peptidase-4; SGLT2, sodium–glucose cotransporter-2; GLP-1, glucagon-like peptide 1; DMARDS, disease-modifying antirheumatic drugs.

The patients used more antihypertensive and psychotropic drugs in the FRID classification as well as other drugs than the controls (*p* < 0.001). When evaluating the ARS scores, a higher mean score was identified in the cases (mean: 2.3 ± 1.9) than in the controls (mean: 1.4 ± 2.2) (*p* < 0.001). The most commonly used anticholinergic medications were trazodone (16.8% vs. 10.8%), quetiapine (12.4% vs. 3.1%), and hydroxyzine (8.0% vs. 3.9%) among cases and controls. The proportions of individuals with moderate (13.6% vs. 7.3%) and high anticholinergic risk (37.2% vs. 24.7%) were greater among the patient group. Polypharmacy, including both the mean number of drugs and the proportion of patients treated with five or more drugs, was greater among the patients than the controls (*p* < 0.001). Table 3 shows the frequencies and proportions of FRID use, polypharmacy, and ARS score.

**TABLE 3:** Frequency and bivariate comparison of the use of FRIDs, classification of the anticholinergic risk scale, and polypharmacy in older hospitalized patients in four hospitals in Colombia.

Variable	Case with fall (*n* = 250)	Control (*n* = 1000)	*p*-value
FRIDs use			
* FRIDs, antihypertensive, at least one (no, %)*	150 (60.0)	458 (45.8)	<0.001
* FRIDs, psychotropic, at least one (no, %)*	154 (61.6)	385 (38.5)	<0.001
* FRIDs, others, at least one (no, %)*	212 (84.8)	683 (68.3)	<0.001
* FRIDs, any, at least one (no, %)*	238 (95.2)	797 (79.7)	<0.001
Medication count			
* Total number of drugs—median (IQR)*	11 (8–16)	9 (5–13)	<0.001
* Polypharmacy ≥ 5 drugs—no (%)*	230 (92.0)	764 (76.4)	<0.001
Risk scale score (ARS scale)*—*mean (DE)	2.25 (1.9)	1.43 (2.2)	<0.001
Anticholinergic risk scale classification*—*no (%)			
* 0 (no risk)*	70 (28.0)	467 (46.7)	<0.001
* 1 (low risk)*	53 (21.2)	213 (21.3)	0.972
* 2 (moderate risk)*	34 (13.6)	73 (7.3)	0.001
* 3 or more (high risk)*	93 (37.2)	247 (24.7)	<0.001

Antihypertensive FRIDs: loop diuretic, alpha 1 antagonist. Psychotropic FRIDS: antipsychotics, antidepressants, and benzodiazepines. FRIDS, others: anticonvulsants, opioids, NSAIDs, and antihistamines.

### Multivariate analysis

In the first conditional logistic regression model, polypharmacy (aOR: 1.9; 95% CI: 1.1–3.4), the use of calcium channel blockers (aOR: 1.3; 95% CI: 1.0–1.8), sodium–glucose cotransporter type 2 inhibitors (iSGLT2; aOR: 4.4; 95% CI: 1.1–16.7), anticonvulsants (aOR: 1.6; 95% CI: 1.1–2.4), typical antipsychotics (aOR: 1.5; 95% CI: 1.0–2.3), atypical antipsychotics (aOR: 1.8; 95% CI: 1.0–3.0), and NSAIDs (aOR: 1.5; 95% CI: 1.1–2.1) were associated with falls during hospitalization. No medication was found to be a protective factor (Table 4).

**TABLE 4:** Conditional multivariate logistic regression evaluating the risk of falls in older hospitalized patients in four hospital centers in Colombia.

	*p*-value	OR (adjusted OR)	95% Confidence interval	
			Lower	Upper
Polypharmacy ≥5 drugs—no (%)	0.014	1.983	1.150	3.420
Being treated during hospitalization				
* *Loop diuretic	0.054	1.376	0.994	1.904
* *Calcium antagonists	0.048	1.372	1.003	1.877
* *SGLT2 inhibitors	0.030	4.403	1.157	16.747
* *Antiepileptic	0.008	1.675	1.141	2.458
* *Typical antipsychotics	0.021	1.598	1.074	2.378
* *Atypical antipsychotics	0.022	1.836	1.093	3.085
* *NSAIDS	0.003	1.596	1.173	2.172
Complication/comorbidity				
* *Delirium	0.002	1.933	1.271	2.940
* *Coronary disease	0.000	0.401	0.271	0.592
* *Stroke	0.020	0.441	0.221	0.880
* *Dizziness	0.006	3.604	1.462	8.882

SGLT2, sodium–glucose cotransporter-2; NSAIDS, nonsteroidal anti-inflammatory drugs.

**FIGURE 1:**
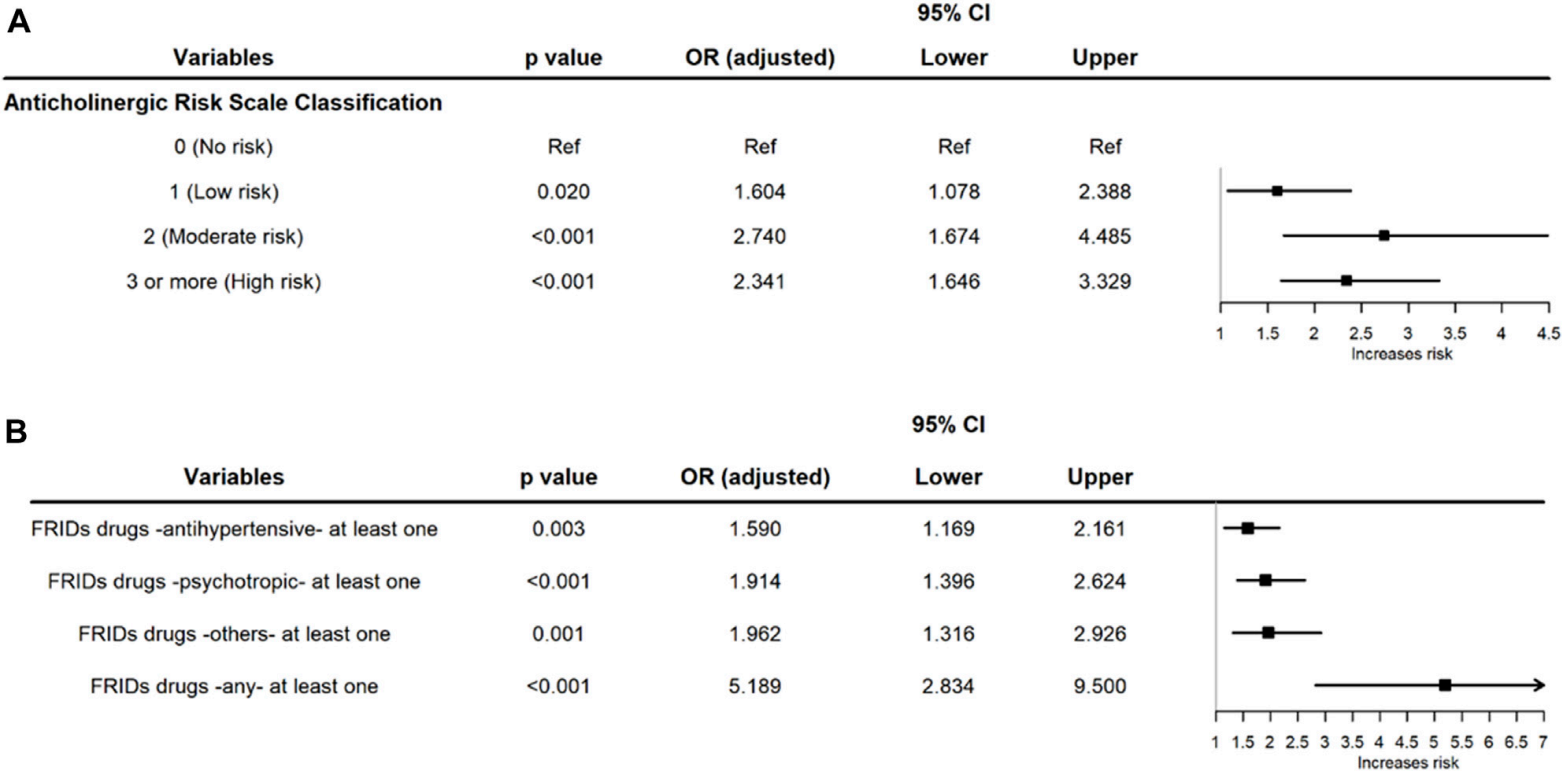
**(A)** Forest plot of the conditional multivariate logistic regression evaluating the risk of falls according to the ARS in older hospitalized patients in four hospitals in Colombia. **(B)** Forest plot of the conditional multivariate logistic regression of the risk of falls in older hospitalized patients taking different FRIDs while hospitalized in four hospitals in Colombia.

In the second conditional logistic regression model, the variables of interest were the ARS score and the risk of falling during hospitalization. From one point of risk (low) upward, the probability of presenting a fall significantly increased if the ARS score was 2 (moderate) or ≥3 (high; see Figure 1A).

In the third conditional logistic regression model, which was constructed according to the use of FRIDs, falls during hospitalization were more common among users of antihypertensive drugs, psychotropic drugs, and other drugs included in this classification. An additional analysis was done by grouping all the FRIDs into a single category, and a significant increase in the risk (aOR: 5.1) of a fall during hospitalization was found in older adult patients (Figure 1B).

## Discussion

This case‒control study in older hospitalized adults found that the risk of falling during the hospital stay was positively associated with the use of FRIDs and drugs with anticholinergic load. This provides evidence for the first time in Colombia of the use of potentially inappropriate drugs in this age group in the context of care within hospitals. These findings should elicit new strategies for the safer use of drugs.

The mean age of the patients and controls included was 77 years. In such a population, with their multiple comorbidities and high risk of frailty (Mudge and Hubbard, 2019), a fall can bring adverse consequences. In a study in Australia, Decalf et al. reported that falls were more frequent in men and in patients older than 70 years, and they occurred mainly in the bathroom and during the night (Decalf et al., 2019). Interestingly, suffering from delirium was also associated with a greater probability of suffering a fall during hospitalization, in line with earlier findings (Hoffman et al., 2019; Wedmann et al., 2019). Therefore, strategies to reduce the risk of delirium include nonpharmacologic measures like early mobilization, adequate hydration, and sleep enhancement.

Moreover, orientation to time and place, and hearing and vision optimization (Oh et al., 2017), as well as trying not to use drugs with an anticholinergic load and others that facilitate its appearance, can also have a positive impact on preventing hospitalized patients from falling and suffering related adverse effects (Tune and Egeli, 1999; Han et al., 2001; Cox et al., 2009; Salahudeen et al., 2016).

Another relevant finding was the association between polypharmacy (≥5 drugs) and an increased risk of falls (Gnjidic et al., 2012), similar to the findings in older hospitalized adults in Brazil reported by Alves–Ramos et al., who established that the association was attenuated after adjusting for comorbidities and increased by the use of FRIDs (Ramos et al., 2023). In Malaysia, Zia et al. recognized that polypharmacy was a predictor of the risk of falling before adjustment for FRIDs (Zia et al., 2017), and polypharmacy lost its significance after the inclusion of FRIDs, so these types of drugs are more significant than the number of active ingredients received by the patient (Gnjidic et al., 2012).

This analysis reflects the multifactorial nature of falls. The causative factors range from advanced age to the presence of comorbidities to the use of certain drugs, especially those with an anticholinergic load, as has been described in outpatients for almost 20 years. This can be attributed to the lack of accompaniment of relatives and nursing personnel, architectural barriers, and safety and patient protection elements (Rudolph et al., 2008). We identified an increased risk of falling (1.6 and 2.3 times greater for patients with low and high anticholinergic load, respectively) in hospitalized adults aged 65 years and older than in those not taking drugs with anticholinergic load. This finding is consistent with several previous studies, such as that of Akgün et al. in the Netherlands, who reported that each one-point increase in the ARS score increased the risk of suffering a fall, with an OR of 1.49 in older hospitalized adults (Akgün et al., 2022), and that reported by Dauphinot et al. in France, who found that higher anticholinergic load increased hospital falls (Dauphinot et al., 2014). This risk is also similar to that found in a study carried out in Colombia in older adults in an outpatient setting who suffered a fall with a hip fracture, in which a higher ARS score increased the probability of falling, which shows an important effect of acetylcholine-mediated peripheral and central nervous system blockage, particularly in older people in both outpatient (Collamati et al., 2016; Bishara et al., 2017) and inpatient settings (Wawruch et al., 2012).

FRIDs (antihypertensive, psychotropic, and others) were associated with the probability of suffering a fall within the hospital environment after multivariate adjustment, as has been identified in ambulatory patients (Milos et al., 2014; Zia et al., 2017; Castaldi et al., 2022; Ramos et al., 2023). It is important to know that the mechanisms of action and adverse reactions of different groups of FRIDs affect adults more than 65 years of age differently, but one thing they share in common is that they enhance the probability of falls, some of which are due to mechanisms such as decreased alertness, the induction of sedation or delirium, and other nonpsychotropic effects such as the generation of orthostatic hypotension or hypoglycemia (Park et al., 2015; Richardson et al., 2015; Nwadiugwu, 2020). However, it is clear that those drugs with an effect on the central nervous system had a stronger overall effect, as well as having individual associations with the risk of falls, as found for antiepileptic drugs and antipsychotics in our multivariate analysis.

The increased use of psychotropic medications, whether typical or atypical antipsychotics, increased the risk of falls in this population, as they were probably prescribed during an episode of psychomotor agitation or during a patient’s hyperactive delirium (Gareri et al., 2014) or in patients who suffered from a comorbidity such as schizophrenia or dementia. Importantly, the use of antipsychotics during an episode of delirium does not truly change the duration, severity, or mortality during the episode, but it does increase the risk of falling during the hospital stay (Neufeld et al., 2016). Antipsychotics can worsen cognitive decline in patients with dementia by altering dopamine levels in the central nervous system; these patients are included in the FRID group and have an anticholinergic load (Rudd et al., 2005; Rudolph et al., 2008; Siafis et al., 2018). The findings of this analysis provide evidence of the undesirable effects of this group of psychotropic drugs in hospitalized adults aged ≥65 and suggest the need to increase precautions for their use, avoid potentially inappropriate prescriptions, carry out greater surveillance and follow-up by healthcare teams, and improve patient safety systems (Prendergast et al., 2022).

The finding in the multivariate analysis of the association between the use of iSGLT2 and falls in adults aged ≥65 is quite interesting because, due to their relatively recent incorporation as antidiabetic drugs, it is striking that they may increase the risk of falling and suffering other adverse outcomes, perhaps related to their effect on glucose, diuresis, or hypotension. There have been reports of falls in older adults who were taking them (Taşdemir et al., 2020), and some authors have suggested deprescribing iSGLT2 and diuretics in older adults with heart failure due to the risk of falls (van Poelgeest et al., 2023). Additionally, iSGLT2 has been associated with altered bone metabolism and increased risk of fractures, so its use in older adults, who more frequently present osteoporosis, sarcopenia, and risk of fractures, should be considered (Ye et al., 2018). Further studies are warranted to explore this possible association.

With these results as a foundation, it should be possible to include older adults who take risky medications in the category of patients at high risk of falling, leading to greater focus on care by healthcare personnel and accompanying family members for these patients.

This study had some limitations, such as not establishing the date of drugs initiation, the dose used, the duration of the prescription during the hospital stay, the laboratory data, or the severity of the clinical conditions, all of which can influence the risk of falls. Some of its strengths are the union of information from four hospital centers in Colombia, a significant sample size of cases and controls, and the innovation of evaluating both the anticholinergic load and the prescription of FRIDs in hospitalized older adults in Colombia.

## Conclusion

Adults aged ≥65 years are more likely to suffer a fall in the hospital if they are given a drug with anticholinergic load or a FRID, as well as if they have some comorbidities, especially delirium, vertigo, or heart disease (coronary ischemia and stroke). There is a clear need to prioritize the implementation of safe prescription criteria in older adults, to carry out a complete evaluation of the effectiveness and safety of the drugs used, and to recognize the potential risk of adverse reactions such as falls. It is important to disseminate these findings among those who care for patients and to carry out new investigations into the relationships that may exist between different medications and the risk of suffering a fall in the hospital.

## Data Availability

The original contributions presented in the study are publicly available. This data can be found here: https://www.protocols.io/private/EC55D8BC983B11EE85760A58A9FEAC02. Further inquiries can be directed to the corresponding author.
