# Supplementary material for: Falls in hospitalized older adults and the use of fall risk-increasing drugs and anticholinergic medications in Colombia: a case‒control study
Source: Front Pharmacol. 2024 Jul 3;15:1369200. doi: 10.3389/fphar.2024.1369200 (PMC11251958; doi:10.3389/fphar.2024.1369200)
Supplement: Supplementary file 1 [file Table1.DOCX]

**Supplementary Table 1.** Anticholinergic Risk Scale (ARS). Taken from Rudolph et al 2008.

| **3 points** | **2 points** | **1 point** |
| --- | --- | --- |
| Amitriptyline | Carbidopa-levodopa | Baclofen |
| Atropine | Entacapone | Cetirizine |
| Chlorpheniramine | Haloperidol | Cimetidine |
| Chlorpromazine | Metoclopramide | Clozapine |
| Cyproheptadine | Mirtazapine | Loperamide |
| Diphenhydramine | Paroxetine | Loratadine |
| Fluphenazine | Pramipexole | Nortriptyline |
| Hydroxyzine | Quetiapine | Olanzapine |
| Imipramine | Ranitidine | Pseudoephedrine |
| Oxybutynin | Risperidone |  |
| Perphenazine | Selegiline |  |
| Promethazine | Trazodone |  |
|  | Ziprasidone |  |
